# Reciprocal Trust as an Ethical Response to the COVID-19 Pandemic

**DOI:** 10.1007/s41649-021-00174-2

**Published:** 2021-04-15

**Authors:** Hui Yun Chan

**Affiliations:** Department of Law, University of Huddersfield, Huddersfield, UK

**Keywords:** Reciprocal trust, Trustworthiness, Ethical practice, Pandemic, COVID-19, Public health

## Abstract

The COVID-19 pandemic has generated a range of responses from countries across the globe in managing and containing infections. Considerable research has highlighted the importance of trust in ethically and effectively managing infectious diseases in the population; however, considerations of reciprocal trust remain limited in debates on pandemic response. This paper aims to broaden the perspective of good ethical practices in managing an infectious disease outbreak by including the role of reciprocal trust. A synthesis of the approaches drawn from South Korea and Taiwan reveals reciprocal trust as an important ethical response to the COVID-19 pandemic. Reciprocal trust offers the opportunity to reconcile the difficulties arising from restrictive measures for protecting population health and individual rights.

## Introduction

COVID-19 continues to be a part of our daily lives. As countries around the world continue to stem the tide of emerging COVID-19 strains, many countries, including Sweden and France, have turned their gaze to Southeast Asian countries as exemplars of pandemic management (Fisher and Choe 2020). Measures undertaken to trace and contain transmissions include fast and vast testing regimes, clear, consistent and streamlined communication and public education and publicity on hygienic practices such as regular hand washing and mask wearing (Partridge-Hicks 2020; Sridhar 2020). These approaches typify some important shared elements of ethical practices fostering trust in the authorities. Authorities, in this context, refer to government official or those entrusted with responsibility to discharge duties to the public. Trust is often associated with doctor-patient healthcare encounters; however, the pandemic has refocused the role of trust in broader social contexts. Indeed, the current research has emphasised the utility and importance of trust in authorities in managing the pandemic (Wong and Jensen 2020; Paek et al. 2008). The rise in campaigns against public health actions such as mask wearing, vaccinations, test and trace programmes, mandatory quarantine and lockdown gestures towards distrust in authorities (Haddad 2021; Safi 2021; Stewart 2020; Picheta 2020; Read 2020). These responses however reveal a deeper concern for trust within the pandemic ecosystem: That reciprocal trust is absent from a diverse range of ethical frameworks. I will explore the relationship between trust and reciprocal trust in the relevant section below. The pandemic ecosystem comprises a complex environment, with various interconnected social, economic, political factors and networks of population and interactions, all of which presents a challenging environment for pandemic relief efforts. Reciprocal trust is an ethically important response to COVID-19 due to a sustained urgency posed by a highly transmissible virus requiring the collective effort from the authorities and the population. These collective efforts necessitate trade-offs from the population (such as movement restrictions), which could be more than in ordinary times, and complex decision-making by the authorities in balancing the different priorities and interests that operate in a pandemic ecosystem. Reciprocal trust thus cushions the harshness of restrictive measures and inconvenience experienced by the population. Inattention to this element consequently widens the gulf of trust to the detriment of population health. This paper aims to broaden the perspective of good ethical practices in managing the COVID-19 pandemic by including the role of reciprocal trust. It will illustrate with examples that engender reciprocal trust drawn from the practices adopted primarily by South Korea and Taiwan and examine how such reciprocity is deployed and negotiated in the COVID-19 pandemic management context. These countries are chosen as exemplars for their successful exemplification of reciprocal trust between the authorities and the population and within the population in curbing the spread of the pandemic. I will demonstrate how reciprocal trust is promoted in the pandemic context supported by appropriate examples drawn from these two jurisdictions in the relevant sections below. It will be clear that prior trustworthy experience is highly likely to support reciprocal trust, which explains the general willingness of the South Koreans and Taiwanese in engaging in voluntary exchanges in view of the restrictions based on previous successes in pandemic (MERS, SARS) managements.

## Reciprocal Trust as an Ethical Response to the COVID-19 Pandemic

### Conceptions and Characterisations of Reciprocal Trust

Research on reciprocal trust features notably in management and organisation studies (Serva et al. 2005; Korsgaard et al. 2015). Reciprocal trust, which is considered to have significant organisational and interpersonal implications, is defined as “the trust that results when a party observes the actions of another and reconsiders one’s trust-related attitudes and subsequent behaviours based on those observations” in a project management team setting (Serva et al. 2005, 625). This characterisation of reciprocal trust indicates that the presence of trustworthiness of one party is likely to foretell the other party’s perception of such trustworthiness that will then lead to ensuing trust and actions that convey that trust. Although the exploratory research is focused on project management settings, the study sheds light on the existence of reciprocal trust in considerably large groups and complex social settings, the dynamic interactions of different factors affecting trust over a course of time and the psychological aspects of trust in relationships which are demonstrated by behaviours and responses that manifest trust or otherwise (Serva et al. 2005, 626, 627). These findings are pertinent to the pandemic context as the pandemic ecosystem is varied, highly complex and liable to many permutations. The authors correctly observed that reciprocal trust requires an appreciation for the role of trust in a relationship, particularly the active process in understanding how trust is reciprocated, gained or lost (Serva et al. 2005, 627, 628; Korsgaard 2018, 14). This observation suggests that the presence of trust or trustworthiness is crucial in forming reciprocal trust and subsequent demonstrations of trust. Reciprocal trust is thus understood as one’s trust that affect the other party’s trust through actions and behaviours that demonstrate the attribute of trustworthiness. This understanding brings to light the self-reinforcing nature of reciprocal trust (Korsgaard et al. 2015, 53), thus amplifying the “trust-begets-trust” paradigm. An example of such self-reinforcing behaviour of reciprocal trust is the positive association between leaders’ trust in the followers in organisations and vice versa (Korsgaard et al. 2015, 54).

Another important characterisation of reciprocal trust is it is a process rather than a “construct” (Korsgaard et al. 2015), similar to Serva and colleagues’ (2005) reference to its dynamic nature that occurs over a period of time. The process continues as long as mutually beneficial outcomes exist and cease to exist where there is no trust (Korsgaard et al. 2015). The processual nature of reciprocal trust indicates the variability of trust levels throughout the interactions and the relationships formed between the parties. Consequently, where trust is felt to be violated, voluntary exchanges between the parties would cease, as evidenced through protests and remonstrations against restrictions in a pandemic context. Arising from this appreciation for its process, reciprocal trust can be characterised as a “bidirectional” occurrence (Korsgaard 2018, 14). The bidirectional aspect of reciprocal trust encompasses continuous “cycle[s] of relationships between trust and cooperation represented by paths from trust to cooperation within persons and from cooperation to trust between persons …with both parties giving and receiving benefits and thus is both a trustor and a trustee” (Korsgaard 2018, 16). The bidirectional nature of reciprocal trust lends weight to the notion that it has a circular effect. The strength of the relationships changes throughout these interactions, as the parties familiarise themselves with the motives, values and interests leading to reviews of the aims of the reciprocal relationship (Korsgaard 2018, 17, 23).

Reciprocal trust is recently represented in the general healthcare literature from the perspective of reciprocal relationships. A functional healthcare relationship possesses attributes that reflect reciprocity, for example trust in the healthcare professionals and reciprocal trust in following the recommendations towards health recovery. A trustor’s perceptions of trustees’ ability, benevolence or integrity, reflected by clinicians’ technical ability, skill or competence, and interpersonal skills thus contribute to trust in healthcare (Peters and Bilton 2018, 333). Consequently, a reciprocal relationship is relevant to the trust framework, while trust as an element in the healthcare relationship can engender reciprocity, thereby strengthening the reciprocal nature of the relationship. Reciprocal relationships in healthcare often embody common aspirations, and shared values of respect and trust that support such relationships (Tumosa 2017). Common aspirations or shared goals enable people to change or cultivate new behaviours to achieve the goal of wellness in their healthcare experience. Reciprocal trust is premised on the acceptance of inherent vulnerabilities within a mutually dependent setting requiring mutual respect and trust to achieve mutually beneficial outcomes. Reciprocal trust can be characterised by various attributes, skills and behaviours ranging from respectful listening and averting presumptions to canvassing patient views and conversations towards improving awareness of what is wrong (Tumosa 2017, 58). These skills, attributes and behaviours are likely to affect the quality of the therapeutic relationship between doctors and patients.

Reciprocal trust is conceivably attained through genuine concern for people, encouraging open discussion, leadership in times of uncertainties or changing circumstances, providing expert advice and responding with empathy to intense situations (Robinson 2016, 10). Reciprocal trust is relational to people and between people, either the authorities or the population in general. The “circular” nature of reciprocal behaviours “serves to grow and sustain the patterns” (Robinson 2016, 3). Thorne and Robinson (1988) advocate for reciprocal trust as necessary in maintaining a functional healthcare relationship, for both the carer and the cared for. This perspective is more nuanced than trust because reciprocal trust signals that “trust from health care professionals fosters trust in health care professionals” (Thorne and Robinson 1988, 786), indicating continuous, conscious actions and behaviours that influence the other rather than a one-directional feature of trust. Reciprocal trust enables us to reconsider the relationship between trust and reciprocal trust in pandemic management, protecting the public from harm and trustworthy communication. It is through reciprocal trust that trust can flourish, where trust instils professional capability, consequently influencing the cared for in handling their illness towards achieving wellness (Thorne and Robinson 1988, 787). In a healthcare relationship, reciprocal trust facilitates a constructive exchange of information to reach decisions appropriate for the circumstances and which are more likely to be accepted by family members (Robinson 2016, 9).

### A Reciprocal Trust Conception in a Pandemic Context

The key conceptions of reciprocal trust drawn from the preceding section are instrumental in presenting a working definition of reciprocal trust for the present paper. Trust is context specific, and so is reciprocal trust. Reciprocal trust, in the context of the pandemic, refers to “a cyclical, reciprocal relationship based on trustworthy actions towards achieving the shared aspiration of population well-being.” This definition of reciprocal trust contains three important attributes. First, I characterise the relationship between the parties, whether between the authorities and the population or within the population as relational and circular in nature. This approach is consistent with the highly complex and interconnected factors and actors existing in the pandemic ecosystem, where the actions of one influence the other. This understanding brings to light the dynamic nature of reciprocity, where it is understood as an ongoing process, requiring a constant negotiation and renegotiation of powers and actions. Consequently, reciprocal trust is facilitative in building and sustaining the relationships between the parties based on a mutual understanding of, amongst others, shared vulnerabilities, while being mindful of the necessary trade-offs in a pandemic. The shared understanding is helpful in facilitating trust or strengthening the bonds between the parties, demonstrated through cooperative or collaborative actions. Reciprocal trust is more likely to flourish if trust exists.

The second aspect of trustworthy actions points to the essential feature of demonstrating evidence of trust in order to promote reciprocal trust. This means that the authorities have to evidence the extent to which they are competent, reliable and honest in fulfilling their distinct role in society, in this case, towards the goal of breaking the chain of transmissions initially, and achieving population well-being in the long term as countries slowly recover from the ramifications of the pandemic. Trustworthy actions suggest how well the authorities resolve the difficulties faced by the population, which will then lead to the population reciprocating that trustworthiness by displaying cooperative responses to the proposed measures. This may require empirical substantiation, but the examples in the sections below offer a persuasive inference that trust is reciprocated where evidence of trust exists in the first place. We will see examples of how feedback (often demonstrated by actions) from the population to various health interventions during the pandemic leads to improvements of the action plans that are implemented, and supported by effective, accessible communications between the parties. Consequently, actions embodying trustworthiness promote reciprocal trust. Actions that cultivate reciprocity in relationships demonstrate that the population is treated as collaborators, rather than passive recipients of advice in a top-down approach *demanding* compliance.

Third, I have adopted the term population well-being to reflect not only the physical and mental health and well-being of the population, but also their social and economic well-being in the broadest sense. The pandemic affects the population’s lived experience in an incredibly challenging way. We are all vulnerable in different ways; however, the pandemic has amplified these vulnerabilities, and which has affected the most disadvantageous population, such as additional burdens on working households, gendered responsibilities and precarious working conditions. The actions taken by the authorities in managing the pandemic must be directed to address these challenges. These actions are premised on an appreciation of the relational and circular effects of the pandemic, where, for instance, if there is a lack of actual relief support, the authorities may be perceived as untrustworthy, resulting in an unwillingness to reciprocate their trust in complying with the pandemic restrictions, leading to a deterioration in the health and well-being of the population.

Reciprocal trust, as understood above, is important in appreciating the dynamics of reciprocity and trust in a pandemic setting, helping us identify actions that are likely to promote or shatter trust. When there is a loss of, or depletion in trust, the relationship is no longer perceived as mutual or reciprocal, resulting in revolt or disengagement from the shared interests and aspirations that are essential in a pandemic. Discontent with the authorities, evidenced from remonstrations to restrictions, suggests that trust in the authorities is withdrawn. Maintaining a relationship of reciprocal trust is necessary in health governance in pandemic management. The characterisation of reciprocal trust reveals that trust is an essential element in promoting reciprocal trust. It becomes necessary to consider the relationship between trust and reciprocal trust in understanding how each of them influences the other and consequently their application in the pandemic.

#### The Relationship Between Trust and Reciprocal Trust

Trust is “the willingness of a party to be vulnerable to the actions of another party based on the expectation that the other will perform a particular action important to the trustor, irrespective of the ability to monitor or control that other party” (Serva et al. 2005; Korsgaard 2018). Trust is thus characterised as unidirectional, with the truster (the party that trusts the other) trusting the trustee (the party who is being trusted or entrusted with something) and acting in a manner consistent with an attitude of trust towards the other. Let us consider the following example of trust: If I disclose a secret to you, I trust that you will not repeat the secret to another and to safeguard that information imparted to you, regardless of my ability to ensure that you do not disclose said information or if you would keep the promise. This means that by disclosing the secret to you, I am being vulnerable and have taken the risk to do so. Let us assume that the interactions continued, and you have displayed reliability and honesty in safeguarding the information, thus heightening my perceptions of your trustworthiness, upon which I continued to engage with you in sustaining our interactions. This example demonstrates reciprocal trust in the relationship. Let us consider the example above from the point of lack of reciprocal trust: I discovered not too long after that you have disclosed the information to the others, leading me to perceive you as being untrustworthy. The interaction between us has consequently changed arising from this change in perception, supported by evidence of such untrustworthiness. Flowing from this realisation, I then consciously disengage from further interactions with you, and would be unlikely to reciprocate your request for trust in future matters involving keeping promises. This shows that there is no reciprocal trust because the initial trust is breached, which then influences my attitude and behaviour towards you and the interaction.

Trust has been characterised as “an ongoing process of building on reasons, routine and reflexivity, suspending irreducible social vulnerability and uncertainty as if they were favourably resolved and maintaining thereby a state of favourable expectation toward the actions and intentions of more or less specific others” (Raaphorst and Van de Walle 2018, 469). This signifies that leap of faith in trust rather than in reciprocal trust where there exists an assessment of trustworthiness of the other based on prior experiences, behaviours and actions. Trust cannot be seen or is not visible in a tangible form. However, trust can be evidenced by trustworthiness arising from another’s ability, benevolence and integrity (Serva et al. 2005, 626; Korsgaard et al. 2015, 53). Korsgaard and colleagues (2015, 49) have described trust as:

an attitude held by one party the trustor toward the other party, the trustee…trust has cognitive, affective and intentional components…the cognitive component reflects the trustor’s beliefs about the character and intentions of the trustee which is based on the trustor’s pre-existing expectations as well as assessments of the characteristics of the other party, the quality of the relationship itself and other situational variables that are likely to influence the relationship.

Trust is thus built on trustworthiness, as vulnerabilities lie in the knowledge imbalance between the truster and the trustee (O’Neill 2018). Consequently, trustworthiness affects reciprocal trust. This means that in transposing this understanding of trust to reciprocal trust relationship in a pandemic, the authorities have to display trustworthy attributes of competence, honesty and reliability: trustworthiness to earn the trust from the population which then enables reciprocal trust to occur. The population would look at evidence of trustworthiness from the actions and conduct of the authorities to then decide if they would like to reciprocate that trust and to sustain that relationship.

Trust is therefore distinguished from reciprocal trust as unidirectional, while reciprocal trust is bidirectional (moving in both directions), where there is a “mutual influence process whereby the trust one party has in the other through its effects on trusting or cooperative behaviour influences the other party’s trust” (Korsgaard et al. 2015, 50). In “simple” trust, only one party is vulnerable and would be made more vulnerable by reposing trust in another, while the other party may have nothing to lose. In contrast, reciprocal trust means that both parties are vulnerable arising from continued engagement in the interactions resulting in potentially loss of anticipated outcome if their goals and motivations are no longer aligned. It is similarly clear from the illustration above that trust promotes reciprocal trust. Trust is often portrayed as static while reciprocal trust is circular and capable of variability in the sense that it is subject to fluctuations depending on the actions of the parties in the interactions through evidence of trustworthiness as a reliable and beneficent party (Korsgaard 2018, 25; Korsgaard et al. 2015). It can be reasonably inferred that trust and reciprocal trust carry practical and significant differences, as they depict important factors affecting the nature, quality and level of trust in a reciprocal relationship.

Another practical and significant difference between trust and reciprocal trust is the presence of gratitude. There is no element of gratitude in trust, while gratitude is present in a reciprocal trust relationship. Gratitude motivates changes in people’s behaviour towards another. This display of gratitude can be overt or covert. Examples of overt gestures of gratitude include express display of appreciation for carers (e.g. participating in nationwide “clap for carers” activity or expressing thank you to these workers) or offering of discounts and free food to essential workers. A covert indication of gratitude may range from behind the scenes effort in ensuring that there is ample supply of personal protective equipment for essential workers, or establishing functional testing centres, which would be reciprocated with higher compliance to these efforts or displaying conduct that demonstrate the willingness to participate rather than remonstrate. In turn, the authorities display gratitude to their participation, rather than taking things for granted or expecting continued patience and sacrifices, by ensuring that restrictions are no longer than necessary. There is a bilateral gesture of gratitude emanating from both parties in pandemic management interventions.

Trust is, despite its differences from reciprocal trust, a prerequisite to reciprocal trust, consistent with the notion of trust begets trust. Without trust, reciprocal trust is unlikely to occur. Without trust, reciprocal trust cannot continue; reciprocity is the ingredient that sustains the trusting cycle in the relationship. This suggests a co-dependency between trust and reciprocal trust. This co-dependency has an important role and practical significance in sustaining reciprocal trust. Reciprocal trust crumbles when there is a perceived violation of trust. It is through trust that reciprocal trust can flourish. Consequently, in a pandemic situation, states have to persevere in establishing trust in order to obtain reciprocal trust from the population. For example, reciprocal trust is expressed by the collective adherence to travel restrictions or face mask wearing. I will next consider more closely the application of reciprocal trust in the pandemic.

### Reciprocal Trust in the Pandemic

Reciprocal trust is important in maintaining and sustaining an ongoing, trusting relationship between the authorities and the population in a pandemic, especially in transforming governmental action plans into collective actions at the population level. Recent research has gestured towards reciprocal trust as a key factor that binds the population and the authorities and amongst population horizontally and vertically, exemplified by the population trusting the information and following the recommendations offered by the authorities as accurate and the authorities trusting the population in actioning those recommendations (Harring et al. 2021). The breakdown in reciprocal trust is evidenced through increasingly high reliance on surveillance measures and enforcement for compliance of pandemic restrictions and vice versa (Harring et al. 2021). The pandemic illustrates shared vulnerabilities, which necessitates competent actions to overcome the difficulties of coping with the pandemic (illness). A pandemic is likened to a disease or illness experienced by individuals who are ill in a doctor-patient relationship, but more widespread and affecting more individuals. The vulnerabilities experienced by people are now increased and not confined to personal experiences of coping with the illness. Reciprocal trust thus has particular significance in a pandemic setting because there is mutual dependence: on one part the compliance with the measures imposed by the authorities and the other, the openness, reasoning and accountability of the government in introducing restrictive measures. In order to function effectively, the relationship between the authorities and the population requires reciprocal trust in successfully managing the pandemic. In the pandemic context, reciprocal trust refers to actions that sustain the relationship between the authorities and the population and amongst the population in breaking the chain of transmissions. Trust is mutual and not taken for granted or demanded; rather, authorities assume responsibility in achieving reciprocal trust from the population in managing the pandemic. The paper will now consider manifestations of reciprocal trust in the pandemic.

#### Examples of Reciprocal Trust in the Pandemic

Let us consider the example of the supply and rationing of face masks during the initial stage of the pandemic where there is a shortage in South Korea leading to public panic. The authorities stepped in swiftly, implementing rationing and shortage supply issues, which demonstrated their competence in handling the shortage (Moon 2020). The authorities, through their competent and reliable actions, provide evidence of their trustworthiness in managing the shortage and alleviating public panic, resulting in compliance from the public in accessing facemasks with cooperation from suppliers in providing facemasks. One of the major successes demonstrated by Taiwan and South Korea is a record of reliability in pandemic management strategies based on previous successful experience in managing infectious diseases such as MERS and SARS. These evidence offer the population a reason to reciprocate the trust. Where the population do not feel assuaged, then the stockpiling is more likely to continue, leading to rejection of further compliance with proposed measures. Competence, honesty and reliability are attributes of trustworthiness (O’Neill 2017, 2018). These attributes speak to the core of supporting trust and subsequently reciprocal trust. The authorities in this example, by being open about the shortage and acknowledging the difficulty in supply and demand but taking steps to remedy the shortfall immediately through restrictions, have allowed the population to appreciate the real situation, resulting in a higher inclination to reciprocate that trust to restore the shortage and for them to acquiesce to the rationing measure until such time that supply returns to normal.

Transparency has often been associated with trust; however, it does not guarantee accessibility (O’Neill 2018). Consequently, it is unlikely to play a role in promoting reciprocal trust. Additionally, transparency in communicating information does not always correlate with voluntary compliance of policies or across all domains (Porumbesco et al. 2017). Openness, on the other hand, means accessibility; and being accessible is “evidence” for which the population can judge the extent of trustworthiness of the authorities and for them to decide if they would like to reciprocate that trust. As O’Neill (2009) rightly observed, “without accessibility, communicative acts fail because they cannot communicate with intended audiences. Some may be unintelligible because intended audiences cannot follow what is communicated: Even if satisfactory as acts of self-expression, they inevitably fail as communication.” As an illustration, the Taiwanese authorities used easy-to-follow, interactive visual communication in extrapolating the meaning, significance and gravity of the pandemic and imparting important messages about daily preventative measures to stop the spread of infection through wearing face masks, hand washing and social distancing that are accessible and intelligible to the population (Hsieh and Child 2020; Lee et al. 2020). The onus is on the authorities to make important information accessible so that the population feel included in the effort to counter the spread of infections and subsequently promote the reciprocal trust of the people. Once the population truly understand their important role in pandemic management strategies, they would then decide whether they would like to participate in the collective effort and to reciprocate that trust. Similarly, if we apply the approach of accessibility in communications regarding preferences for one course of action over the other in managing the pandemic, such communications must be accessible to the people, so that they can understand what is the adopted strategy, why it is selected and what are the implications. Authorities therefore must not be economical with the truth. Being accessible in this sense promotes reciprocal trust.

How do authorities reciprocate the (presumptive) trust from people in handling personal information arising from test and trace systems? The ability to collect information rightly raises privacy concerns, putting authorities in situations of power with identifiable and potentially sensitive information of the public (Zastrow 2020). An ethical response is an accountable, assurance of privacy, where, despite the potential of identifiable information arising from test and trace applications, the authorities should offer explanations on how this collected information is used, and the safeguards installed to protect such information in the public domain. This approach not only reflects openness, but also treats population as collaborators with respect, illustrating the continuous actions needed in sustaining reciprocal trust. The willingness to trade-off privacy for public health is evident in the South Korea and Taiwan approaches (Thompson 2020; Lee et al. 2020; Marszalek 2020). These approaches include efficient, centralised communication channels, effective leadership, cohesive collaborations with all levels of government, well-prepared and adaptive infectious diseases plan and stringent test and trace system. These trust-generating actions lead to a higher level of population participation in proposed restrictive measures such as quarantine, travel restrictions or stay-at-home instructions, physical distancing and face mask wearing. Where there is a perceived discrepancy between competing interests or assumptions that people are not prioritised, as in the example of prioritising economic safety over population health (Mainous 2020), reciprocal trust cannot prevail. Reciprocal trust cannot prevail in such circumstances because the authorities’ lack of trustworthiness in taking actions to prioritise public health sends a message to the population that engagements with any proposed measures to contain the spread of infections are not crucial, consequently leading to behavioural changes and a business-as-usual mindset. It is reasonable to postulate further that the population might form the perception that they have to take matters into their own hands to protect themselves because they could no longer trust that the authorities have their best interests in recommended policy actions. Where there is a lack or absence of reciprocal trust, the population’s level of participation in pandemic management measures will either plummet or become disengaged, leading to social and healthcare costs, such as increased hospitalisations, death and long-term mental health consequences. The socially disadvantaged may be more likely to experience the brunt of social and economic consequences, which will then spiral to a lower level affecting their longer term recovery.

Reciprocal trust has a circular effect with the potential to shape the lived experience of people under pandemic conditions. People are already experiencing transformations in how they socialise, work, communicate, shop, travel, sleep, live and make decisions, big or small. Consequently, reciprocal trust between authorities and the population and amongst the population is indispensable in pandemic management, not least because the pandemic entails social, personal and economic consequences but also in dealing with further unknowns that are likely to develop along the continuum of the pandemic. Authorities have to confront both cognitive and psychosocial factors affecting the population’s inclination to comply with restrictions (Prati et al. 2011; Hendy 2020). For example, South Korea’s success in curbing the COVID-19 pandemic is far from an overnight effort but a sustained endeavour derived from previous pandemics such as MERS and SARS where shortcomings foregrounded by public criticism of the mishandling of these pandemics led to the authorities revamping their approaches towards managing infectious diseases (Ragavan 2020; Thompson 2020). Pandemic response measures demonstrate that to promote reciprocal trust, the authorities with clear responsibility in making decisions in times of public health crisis must convey a clear message in taking actions that protect public health while accommodating population needs, thus gaining the trust of the people, who are then more likely to collaborate and reciprocate through participation and behavioural changes for temporary inconvenience (Kim 2020; Choi 2020; Park 2020; Fouser 2020). Reciprocal trust thus generates more willingness of united actions (Siegrist and Zingg 2014; Roy 2020) and galvanises the population to act in solidarity against a common COVID-19 threat (Libal and Kashwan 2020).

The process of achieving reciprocal trust is dynamic, not free from values, and is liable to constant renegotiation between the stakeholders as the trajectory of the pandemic develops. Values that are relevant to reciprocal trust in a pandemic context include honesty, reliability, competence and citizenship, which highlight the potential conflict of competing interests between the authorities and the population vis-à-vis implementing and complying with temporary restrictive measures. An example to illustrate the need for constant renegotiation in a relationship that embodies reciprocal trust is implementing and lifting restrictions on things that you can do and cannot do. The public would want to know the reasons and the length of time for which the restrictions are imposed, and the authorities, having the power to do so, must execute these measures well in a reliable and competent manner. There are two key approaches exemplified by countries that have successfully managed the pandemic that contribute to promoting reciprocal trust: psychological safety in the population generated by effective leadership; and clear, consistent public health communication resulting in reciprocity, cooperation and collaboration for the mutual benefit of population and authorities.

#### Reciprocal Trust Between the Authorities and Population

Reciprocal trust underpins the success of key strategies such as rigorous mass testing, quarantine, facemask wearing and other measures due to the trusting relationship that exists in collaborative actions in managing the pandemic. Psychological safety is an influential feature to achieve reciprocal trust in managing population behaviour during pandemics. The pandemic generates various health and psychological anxieties, ranging from fear of being infected by the virus to economic security and restricted social movements, consequently creating various emotional and behavioural responses (Sauer et al. 2020). Creating psychological safety in the population during the pandemic is similar to attempts at regulating their emotional experiences in navigating their daily, lived experiences that are drastically transformed during a pandemic. There are psychological and behavioural consequences such as depression during pandemics, where the population faced limited opportunities in restoring emotional and psychological resources due to competing demands (Restubog et al. 2020). Consequently it becomes important to maintain these emotional experiences in order to minimise adverse emotions that give rise to depression or other negative psychological responses. Psychological safety is predominantly popular in the fields of organisational culture, behaviours and management (Schein and Bennis 1965; Frazier et al. 2017). Psychological safety is characterised as a method to minimise anxiety or the cognitive state of workers within organisational cultures towards creating an environment that encourages positive changes in engaging with work (Schein and Bennis 1965; Frazier et al. 2017). Anxiety, which could arise from vulnerability and lack of trust, creates low psychological safety (Frazier et al. 2017). Other factors that affect a worker’s level of psychological safety include leadership, interpersonal relationships, group dynamics and norms, with positive correlations between psychological safety and good relationship with leaders (Frazier et al. 2017, 117, 140). Recently, the concept of psychological safety has been applied in the context of promoting continuity of learning at schools during the pandemic, where school leaders are encouraged to harness positive psychological safety amongst workers so that workers may effectively respond to changing learning and teaching ways during crisis times (Weiner et al. 2020).

Although the concept of psychological safety is primarily contextualised in the workplace environment, it has relevance to pandemic management, particularly the leadership aspect in demonstrating the relationship between the management (e.g. authorities, governments, broadly construed) and those who are being managed (the population). In considering the factors that promote workers’ engagement with their roles at workplaces, it is similar to identifying important traits that support population engagement with collective efforts advocated by the authorities. The authorities, as the object of and foundation for trust for the population, would take care to support positive psychological safety in the population through trustworthy actions, with the aim of promoting reciprocal trust. For example, in a healthcare setting, care providers “can create trust by providing situational normality or structural assurances” within complex organisational structures and systems (Peters and Bilton 2018, 334). Healthcare providers often symbolise norms and values that represent trustworthiness, amongst others in realising their responsibilities of care to patients. Similarly, the authorities manifest certain values and interests in exercising their responsibility that the population have come to expect in a competent manner in the interest of the public. This aspect is relevant in generating various positive or negative psychological responses, such as confidence or fear and minimise negative emotional responses arising from uncertainties and accept these uncertainties despite the lack of control or knowledge (337).

It is reasonably postulated that the authorities initially have a “reservoir” of trust that are implicitly reposed in them by the population in fulfilling their functions and discharging their responsibilities to the population in ordinary times as well as in times of crisis. Generally, even if the population do not completely trust the authorities (as is normal in most democratic societies), there is a minimum level of trust in the authorities that they will do the right thing during a health crisis. Therefore, even if they may hold some distrust towards the authorities, they may be willing to give them the benefit of doubt and to trust them, with ensuing behaviours adjusted as the interactions continued, and whether they would reciprocate their trust. This phenomenon is consistent with the notion that trust is not a given but must be earned and sustained in order to stimulate reciprocal trust. The authorities usually draw from the implicit reserves of trust, until such reserves are depleted for which the population may not reciprocate their trust. The authorities would be wise to continue adding to the reservoir of trust prior to its depletion through trustworthy actions. Where the population perceives trustworthy actions, they will reciprocate their trust, especially when there are positive outcomes arising from effective pandemic management. A deficit in trust is unlikely to promote reciprocal trust.

The authorities ought to be mindful of the evidence of change in the relationship with the population in the process of promoting reciprocal trust. While most public services are perceived as trustworthy, citizens have lower trust in public administrative bodies or bureaucrats, possibly influenced by their experience in the delivery of public services or engaging in the process and their own attitudes to authorities (Raaphorst and Van de Walle 2018, 470). Trust or distrust in public authorities is manifested in various ways, from voicing their frustrations online or in physical protests and withdrawing from participating in governmental arrangements (such as refusing to send children to schools or anti vaccinations) (Raaphorst and Van de Walle 2018, 471, 472). Likewise, the authorities’ trust in citizens is demonstrated through the level of public monitoring and enforcement of actions. Translated to the pandemic setting, this would apply in the context of compliance with essential travel only rules or face mask wearing.

The pandemic tests the boundaries of vulnerability and mutuality of interests. It exposes the weaknesses in the existing pandemic management infrastructure, and the tensions that arise from competing priorities, while casting the light on shared interests and purpose. The growing scientific evidence surrounding COVID-19 calls for a certain amount of leap of faith between the authorities and the population due to the many uncertainties in managing the pandemic. These uncertainties deplete resilience and resemble the antithesis of safety and assurance (Killgore et al. 2020). There is no reciprocity when people are uninformed or where information is suppressed, and where people’s questions remain unanswered. In this climate, psychological safety is breached as fear is not assuaged; uncertainties grow, culminating in refusal to comply with measures that are directed at curbing transmission of infections (Melimopoulos 2021).

Replacing fear with genuine reassurance is therefore critical in various key stages of pandemic management. Such assurance can be derived from proactive, concrete pandemic preparedness actions that are accessible to the population so that reciprocal trust can be generated and behavioural changes promoted. These actions range from functional testing centres being in place, sufficient supply of personal protective equipment and economic packages to support temporary unemployment or costs for quarantines (Lee et al. 2020). Such measures demonstrate that the authority prioritises the people in coping with the disruptions to their lives due to the pandemic (Marszalek 2020). An example to illustrate this is the infrastructure readiness in South Korea and Taiwan where pandemic management resources are in place in advance of public announcements or dissemination of key information (Choi 2020; Ahn 2020). This infrastructure readiness is especially vital in assuring the public about the state of transmission unfolding before their eyes, what is being done and what is required from them, leading to reciprocity in trust and actions. When the public perceived that their expectations on pandemic relief resources were largely met, such as the readiness of quarantine support and information, they are more likely to reciprocate their trust through compliant actions. Public anxiety is likely to be quelled or minimised because of the authorities’ openness which in turn promote public confidence in the adopted approach. South Korea implemented strict, mandatory quarantine and fines or deportation for violations (Thompson 2020). When the public perceived that breaches to restrictions were not tolerated, for example in the case of health officials enforcing quarantine monitoring, it shifted people’s psychological and behavioural responses (Yeh and Cheng 2020). These constructive measures instilled reciprocal trust in the governance relationship, unlike in some countries where confidence in the authority is low compared to South Korea and Taiwan (McPhillips 2020; Fancourt et al. 2020; Helm 2020; Hsieh and Child 2020). It is clear that where the population has confidence in the infrastructure preparedness in managing the pandemic, there is a higher likelihood of participation and collaboration from the population in the collective effort to contain the pandemic.

Clear, consistent, timely, accurate and open communications contribute to population assurance and psychological safety, which translates to promoting reciprocal trust. Clear communication is related to competency which encompasses effective messaging and listening skills in responding to the ever-changing situation and judging the best course of actions to take. These may seem elementary but are especially crucial in supporting reciprocal trust, leading to a sense of solidarity in managing the pandemic. Truth telling engenders reciprocal trust and thus cannot be underestimated. The proliferation of information across multiple social media platforms enabled a comparison of infection rates and deaths with other countries, which led to confirmation or doubts about the veracity of the advice and appropriateness of measures taken, subsequently promoting or hindering compliance with safety measures (Wong and Jensen 2020). It is hence essential to avoid sudden unexplained reversals of approaches or inexplicable reasoning for adopting particular strategies. Timely communication requires competency in conveying information in a way that manages public “panic and fear” and the preparedness to request the public for help in reciprocating the actions in confronting the virus (Ragavan 2020; McPhillips 2020). Increased voluntary cooperation from the population in turn reinforces authorities’ effort.

Recent research similarly support the approach of providing timely, accurate and contextualised information to the population to allay the anxiety arising from the uncertainties of future dangers, including information about “the expected outcome of different approaches, vaccine development and prevalence of infections” followed by clear justifications for adopting specific approaches (Blanco et al. 2020, 2758). Comparisons tend to be made where there are variable approaches and the chosen course of action should be clearly explicated to enable the population to understand the foundation of these choices between competing socio-economic interests. An understanding of these choices is likely to cultivate social support and solidarity in surviving individual sacrifices in the pandemic (Blanco et al. 2020). Actions that promote reciprocal trust are likely to enable health authorities to gain the cooperation from marginalised communities in identifying potentially vulnerable groups and their specific needs through tailored communication of risks and public health strategies (Henderson et al. 2020). This includes paying attention to the marginalised societal groups including single parents, and those in the lower socio-economic and migrant groups in managing the pandemic.

Effective, competent leadership promotes psychological safety in the population and engenders reciprocal trust in the relationship. The state shoulders the main responsibility in organising and coordinating containment efforts, owing to its resources and access to information. Consequently states have to persevere in establishing trust in order to obtain reciprocal trust from the population. An example of an effective leadership that translates to reciprocal trust is South Korea where lockdown and the attendant socio-economic consequences have been averted. Taiwan similarly demonstrated centralised leadership, voluntary participation and effective network of monitoring and coordination (Yeh and Cheng 2020; Lee et al. 2020; Hsieh and Child 2020). Positive tangible outcomes arising from proactive strategies lead to increased collaborations that contain the tide of infection. An example is the effective intervention in addressing facemask shortage which calmed population anxiety (Moon 2020; Ahn 2020). This may seem an oversimplification of factors, given other important contributions that shaped compliance with restrictions (such as financial, social and psychological incentives) (Choi 2020; Yeh and Cheng 2020; Prati et al. 2011); however, perceptions of competent, coordinated leadership are likely to be clear indicators of strengthening reciprocal trust between the authorities and the population. The circular effect between effective performance of pandemic management and reciprocal trust is significant in gauging and shifting public behaviour. Consequently, an agile and adaptive leadership in contrast to lagged, ill-prepared management creates desired collaborative effects (Moon 2020). Reciprocal trust can be seen as severely lacking in behavioural responses that manifest resistance towards measures such as mask wearing, lockdown and quarantining and objections to vague and inconsistent implementation of rules. An open, interactive approach between the authority and the population towards building consensus on solutions (Hsieh and Child 2020) fosters public reciprocity, thus demonstrating a shared purpose and collective endeavour in breaking the chain of transmission (Wilson 2020; Cairney and Wellstead 2020). These attributes contribute to maintaining reliability of the authorities (Henderson et al. 2020) in strengthening reciprocal trust.

Comparison is never far from the gaze of the population as the world becomes more connected than ever before. The World Health Organization is perceived as the guiding beacon in responding to public health threats in its role in preparing for, preventing, protecting against and detecting risks of outbreak during health emergencies. The availability of international standards in managing infectious diseases, such as supporting technical guidance (WHO 2020) and the International Health Regulations, enables a sense of assurance for the population when the authorities are regarded as following established practices, thus influencing population perceptions of leadership, which would be more likely in leading to a higher likelihood of compliance. Where actions produce desirable outcomes, they increase reciprocity because trust levels remain high. The nature of the pandemic lends itself to heightened vulnerability arising from various risks to life and curtailment of rights. Such reciprocity may be tempered by time, culture and specific contexts; however, reciprocal trust is underpinned by an acknowledged but potential vulnerability and faith in the authorities taking appropriate actions. Such actions include effective coordination between local authorities and other stakeholders—exemplified by hospitals arranging for referrals “even in the absence of legal framework” (Choi 2020). This demonstrates increased reciprocity from the existing level in the relationship towards achieving a zero-outbreak target.

#### Reciprocal Trust Amongst the Population

Reciprocal trust between the authorities and the population can translate to reciprocal trust amongst the population, from individuals to communities. Reciprocal trust that exists between the authorities and the population is dynamic and has the galvanising effect on the population, which continues to influence the attitude and behaviours of the population. While reciprocal trust at the authorities-population is not necessarily a prerequisite to reciprocity at the population level, pandemic management strategies are more likely to be collaboratively carried out, with resourcing needs identified at each level of the population, consequently enabling a virtuous cycle of exchanges. The reciprocal trust that initially moves vertically (between authorities and population) will then extend horizontally amongst the population, as the population is now bound by shared goals, common interests *and* supportive strategies to manage the pandemic together. Taiwan is a case that demonstrates collaborative efforts amongst the population (Schwartz and Yen 2017). There is less of an “I” and more of a “we” and “us” in complying with the temporary restrictive measures. Reciprocal trust within the population is important to galvanise the different segments of the society with varying needs and vulnerabilities. Collaborative participation enables a “better understanding of local conditions, vulnerabilities and capacities and better allocation of resources” (Schwartz and Yen 2017, 127), resulting in heightened reciprocity amongst the population. Reciprocity amongst population occurs in trusting that people will comply with the restrictions, such as isolating upon arrival from specified countries supported by monitoring and enforcement (New Zealand), wearing masks and physical distancing (South Korea, Taiwan, Singapore). Similarly, stockpiling essential items and implementing rationing measures are less likely to occur (Moon 2020). The presence of reciprocal trust can be seen from people taking increased responsibility in reporting and updating information about their health conditions through digital reporting of COVID-19 symptoms, and efficient contact tracing supported by robust technological infrastructure (Choi 2020). This will be effective where the broad phase of reciprocal trust between the authorities and population is satisfied.

Public support is central in ensuring compliance as it is impracticable to expect authorities to have the capability to manage quarantined population (Choi 2020). Reciprocal trust potentially creates a more engaged population to act consistent with restrictive measures. It can elevate common interests over individual rights. For example, the active deployment of test, track and trace can only work with reciprocal trust and actions from the population. Reciprocity is accordingly highly relative to the success of public health measures. The strategies of “be right, be first, build trust, express empathy and promote action” are steps that instil reciprocity (You 2020). These steps demonstrate the trustworthiness of the authorities which in turn generate reciprocal changes in the population in their attitude to and interactions with the authorities in the course of the pandemic. As COVID-19 mutations continue to occur, combined with the imminence of successive waves of infections, it is essential and timely to support reciprocal trust by implementing effective approaches, and inform policy decision-making for current and future pandemic preparations.

## Conclusion

COVID-19 has increased the significance of reciprocal trust in pandemic management. It brings to light vital ethical practices that challenge current ways of thinking about the relationship between the authorities and the population, and amongst the people. It prompts us to reconsider the role of reciprocal trust in our ethical view through the experience of dealing with COVID-19. Psychological safety encourages reciprocal trust amongst people and vice versa, where the population and the authorities play their distinct roles in the reciprocal relationship while offering the opportunity to reconcile competing interests. The authorities need to appreciate the dynamic nature of the social agreement between them and the population, underscoring the significance of reciprocal trust as a continuous process, requiring a constant negotiation and readjustments of actions. While not all strategies can be replicated owing to differences in the socio-legal structures, active steps that support reciprocal trust are necessary in whatever measures taken by the authorities. These are illustrated through steps that represent a coordinated, collaborative action, and approaches that enable prompt feedback, and flexible and open responses. Reciprocal trust can help gauge public view of actions taken and how to improve them in containing COVID-19 transmissions. Clarity in pandemic management strategies and consistent and streamlined actions strengthen reciprocal trust. Early, proactive measures underpinned by reciprocal trust can pre-empt harsh, successive lockdowns, which, over a period of time, create weariness and distrust, detrimentally affecting compliance. Consequently, strengthening reciprocal trust is one of the lessons learned from this pandemic.
